# Research on Shrinkage in Lithium Slag Geopolymer Mortar: Effects of Mix Proportions and a Shrinkage Prediction Model

**DOI:** 10.3390/ma18204766

**Published:** 2025-10-17

**Authors:** Lei Wang, Gao Pan, Cai Wu, Sidong Xu, Daopei Zhu

**Affiliations:** 1 School of Software Engineering, Jiangxi University of Science and Technology, Nanchang 330013, China; 2 School of Civil Engineering, Hubei Engineering University, Xiaogan 432000, China; 3 Yichun Lithium New Energy Industry Research Institute, Jiangxi University of Science and Technology, Yichun 336023, China

**Keywords:** lithium slag geopolymer mortar, shrinkage, mix proportion, TG-DTG analysis, MIP test, porosity ratio, shrinkage prediction model

## Abstract

Lithium slag (LS), a solid waste generated during lithium smelting, exhibits significant potential for geopolymer preparation. However, the high shrinkage of lithium slag geopolymer mortar (LSGM) severely restricts its engineering application. Currently, research on the effects of mix proportions (GBFS-LS mass ratio, water–binder ratio, and binder–sand ratio) on LSGM’s shrinkage, and the correlation between shrinkage behavior and microstructures (pore structure and thermal behavior), remains insufficient. Additionally, there is a lack of targeted shrinkage prediction models for LSGM. To address these research gaps, this study systematically investigates the shrinkage characteristics of LSGM and develops a modified prediction model. Thermogravimetric analysis–differential thermal gravimetric analysis (TG-DTG) results show that a lower GBFS-LS ratio promotes the formation of dense sodium-alumino-silicate hydrate (N-A-S-H) gels. Meanwhile, mercury intrusion porosimetry (MIP) tests demonstrate that optimizing the water–binder ratio and binder–sand ratio refines the pore structure of LSGM, where the average pore size is reduced from 53.5 nm at a GBFS-LS ratio of 3 to 28.75 nm at a GBFS-LS ratio of 1.5.Quantitatively; compared with the group with a GBFS-LS ratio of 3, the 90-day shrinkage strain of the group with a GBFS-LS ratio of 1.5 decreases by 25.8%. When the water–binder ratio decreases from 0.57 to 0.27, the 90-day shrinkage strain reduces by 36.7%; in contrast, increasing the binder–sand ratio from 0.21 to 0.39 leads to a 39.8% increase in 90-day shrinkage strain. Based on the experimental data and the fundamental framework of the American Concrete Institute (ACI) model, this study introduces mix proportion influence coefficients and constructs a novel shrinkage prediction model tailored to LSGM. The coefficient of determination (R^2^) of the proposed model exceeds 0.98. This model provides a reliable quantitative tool for the mix proportion optimization and engineering application of LSGM.

## 1. Introduction

With the transformation of the global energy structure to clean energy [1], lithium resources, as the core strategic material of the electric vehicle and energy storage industry, have seen their mining and smelting scale dramatically expanded [2,3]. According to statistics, for every ton of lithium produced, about 200 tons of waste rock and tailings are produced [4,5]. The global lithium industry generates hundreds of millions of tons of solid waste every year [6]. However, due to the lack of an efficient resource recovery path, the solid waste of lithium slag produced in the process of lithium smelting faces long-term environmental risks, such as storage area and heavy metal leaching pollution [7,8].

As a new type of nonmetallic material, geopolymer is formed by the depolymerization and condensation of alumino-silicate precursors under alkali excitation [9,10,11]. Compared with ordinary Portland cement (OPC), it has the characteristics of salt, alkali, acid, high-temperature resistance, and extremely high mechanical strength (compressive strength: 35–60 MPa; flexural strength: 6–10 MPa) [12,13,14]. Many scholars believe that it has the potential to be a green alternative to OPC [15,16]. Moreover, the raw materials of geopolymer are industrial by-products or other low-cost alumino-silicate materials [17]; notably, the environmental impact of geopolymers is less than that of ordinary Portland cement (OPC), which accounts for 5–8% of global greenhouse gas emissions [18,19].

However, through the study of geopolymer, scholars have found that geopolymers exhibit poor volume stability [20], and their drying shrinkage is much greater than that of ordinary cementitious materials—two issues that significantly limit their application in engineering practice [21,22]. Previous studies have found that the high shrinkage of geopolymer is mainly related to capillary pressure, surface energy change, low-density C-A-S-H gel, and the lack of Ca(OH)_2_, Aft, and AFm crystal phases [22,23]. The alkali-activated material has more mesoporous pores than the cement mixture [24,25]. At low relative humidity (RH < 60% [26,27]), a large amount of water loss from the pores of the binder, common in geopolymer systems with a water–binder ratio of 0.25–0.55 (e.g., blast furnace slag or fly ash-based geopolymers [24,28,29]), causes capillary stress, resulting in shrinkage [30]. The Ca/Si ratio of the hydration products (e.g., C-A-S-H gel [31,32]) in alkali-activated materials is higher than that in OPC, which leads to the formation of more low-density C-A-S-H gels [24]. Some scholars studied the shrinkage performance of geopolymers under different alkali equivalents and found that the higher the alkali equivalent [33,34], the greater the shrinkage rate. Some scholars have also used high-temperature curing and film coating curing to reduce the shrinkage of geopolymers [35].

In terms of material optimization, geopolymers can incorporate industrial wastes like fly ash and silica fume, both with finer particles to replace some precursors [36,37,38]. This incorporation alters the Ca/Si ratio of C-A-S-H gel in the matrix. At the same time, it densifies and refines the pore structure of the gel phase. These changes work together to limit water evaporation and reduce shrinkage [39]. The attainment of a densified pore structure in geopolymers is primarily accomplished by the diminution of alkali concentration and modification of the silicate modulus, which serves to retard the rate of the alkali activation reaction [32,40,41]. A porous water-absorbing aggregate can be added to reduce the capillary stress caused by water evaporation [42]. At present, the research on the shrinkage of geopolymers mainly focuses on the geopolymers based on fly ash and GBFS [43,44,45]. As a solid waste with pozzolanic activity, lithium slag is used as a cementing material for cemented tailings backfill [46,47], an admixture for concrete [48], and a precursor material for geopolymers [49,50,51]. However, there are few studies on the shrinkage of lithium slag geopolymers.

In the experiments presented in this paper, experimental groups with different levels of multiple factors (GBFS-LS ratio, water–binder ratio, cement–sand ratio) were established. Through the comprehensive analysis of TG-DTG, MIP experiments, and shrinkage properties, the effects of the material distribution ratio on gel phase composition, pore properties, and shrinkage stress were systematically analyzed. Finally, based on the basic function of the American Concrete Institute (ACI) model, the ratio influence coefficient is introduced to establish a shrinkage prediction model of lithium slag geopolymers based on component parameters.

## 2. Materials and Methods

### 2.1. Raw Material

**Figure 1:**
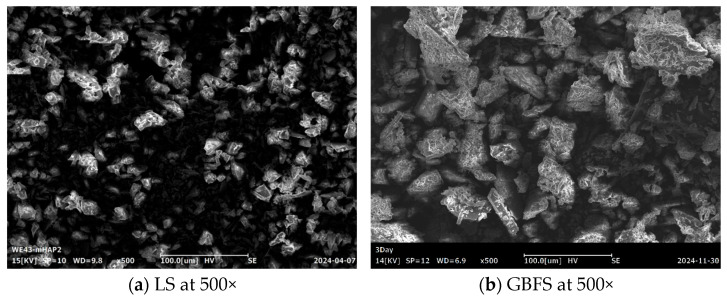
The microstructure form of the raw materials.

**Figure 2:**
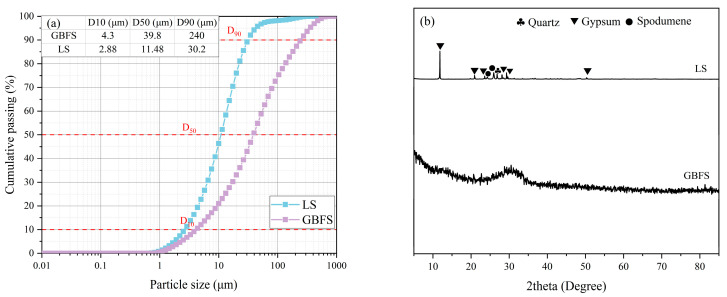
(**a**) Raw material particle size and (**b**) XRD patterns of LS and GBFS.

The alkali excitation materials used in this study are mainly industrial waste lithium slag (LS) and blast furnace slag (GBFS). LS is from Yichun Ganfeng Lithium Group Co., Ltd. (Yichun, China); GBFS comes from Henan Jiewei Environmental Protection Material Company (Henan, China). Figure 1 introduces the microstructure of LS and GBFS through FE-SEM images (ZEISS Sigma 300, Zeiss in Jena, Germany). The fine aggregate used in this study is quartz sand with a mesh size of 80–120. The particle size distribution of these materials was analyzed with a laser particle size analyzer, as shown in Figure 2a. Figure 2b shows the mineral phases of LS and GBFS obtained through XRD testing. The X-ray diffraction (XRD, ARL EQUINOX Pro, Waltham, MA, USA) pattern of GBFS is characterized by a broad amorphous halo, which originates from its dominant glassy phase. Minor crystalline phases are also present. The oxide components of GBFS and LS were analyzed by flame atomic absorption spectrophotometer (FAAS, PinAAcle 500 Flame, Waltham, MA, USA). The experimental results are shown in Table 1.

Sodium hydroxide (NaOH) with purity greater than 99% is obtained from Henan Xiong Chemical Co., Ltd., Xinxiang, China. Silicic acid sodium solution (26.2% silica, 8.3% sodium oxide, 65.5% water) obtained from Henan Maiyuzou Chemical Construction Co., Ltd., Xinxiang, China is used to prepare the alkali activator. First, calculate the amount of NaOH based on a modulus of 1.2: For every 1000 g of Na_2_SiO_3_ solution, 184 g of NaOH particles should be added to reduce the original modulus from 3.2 to 1.2. Pour the Na_2_SiO_3_ solution into a plastic container first, then slowly add the NaOH particles. Use a JJ-5 planetary mixer to stir at 300 rpm for 5 min until the NaOH is completely dissolved. After stirring, place the solution in an environment at 25 °C and let it stand for 24 h to cool to room temperature and eliminate air bubbles.

### 2.2. Methods

#### 2.2.1. Mix Proportion of Geopolymer

The alkali equivalent and modulus of the alkali activator in this experiment were 5% and 1.2, respectively. For the experiment, ten groups of mixtures were prepared, where four levels of groups were set by the control variable method for three factors: the ratio of the mass ratio of GBFS to LS (*R*_G/L_ range: 1.5–3), water–binder ratio (*R*_W/B_ range: 0.27–0.57), and binder–sand ratio (*R*_B/S_ range: 0.21–0.39). The mixing ratios for each group are shown in Table 2. The ratios in Table 3 represent the materials required to make 3 samples.

#### 2.2.2. Preparation Sample

**Figure 3:**
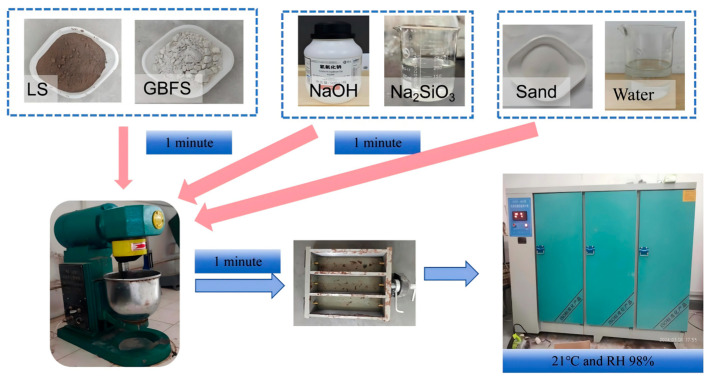
The preparation process of lithium slag LSGM specimens.

Before the experiment, in order to avoid the influence of alkali activator temperature on the experiment, it is necessary to prepare and cool to room temperature in advance. According to the experimental protocol, first weigh the required amounts of mineral raw materials (LS, GBFS), sand, and water. As shown in Figure 3, first, GBFS and LS are added to the blender for 30 s, and then the alkali activator is added to the blender for 1 min. Then, add the remaining water and quartz sand in turn and stir for 1 min. After the raw materials are mixed, the geopolymer mortar is quickly poured into a special mold of 40 mm × 40 mm × 160 mm. Before pouring, a release agent should be applied to the inner wall of the test mold to facilitate the removal of the mold. Then, vibrate on the shaking table for 1 min. Discontinue vibration upon the formation of continuous tiny bubbles on the surface. Use a spatula to smooth the surface. Then cover the test block with a film and place it in a constant temperature and humidity curing chamber for curing (at 21 °C with a relative humidity of 98%). After 24 h, remove the mold and then place the sample back into the curing chamber to continue the curing process under the same conditions.

#### 2.2.3. Shrinkage Test

**Figure 4:**
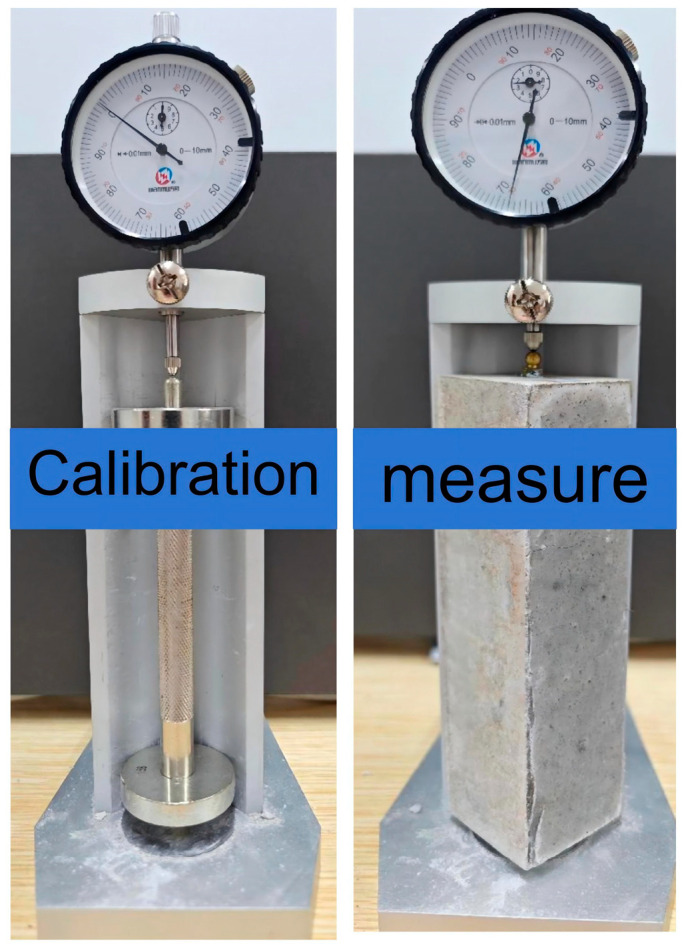
Shrinkage experimental facility.

As shown in Figure 4, in order to fully measure the shrinkage rate of the LSGM throughout the entire process, the length comparator is used to determine the change in length of the experiment according to the standard JGJT70-2009 [52]. The measuring instrument used is an Ames dial with an accuracy of 0.001 mm. Three samples are measured for each group, and the average value is taken. After demolding, the initial length (*L*_0_) was measured. Starting from the release time, the length (*L*_t_) of the samples is measured on the t-th day. *L*_d_ is the length of the two copper nails of the sample. To mitigate the environmental impact on the test specimens, each measurement shall be completed within 3 min. Immediately after the measurement, the specimens shall be returned to the curing chamber (at 21 °C with a relative humidity of 98%). The calculation method of the shrinkage rate is as follows:

(1) $$\varepsilon_{t}=\frac{L_{0}-L_{t}}{L-L_{d}}$$

#### 2.2.4. Microstructure Analysis of Geopolymer

TG-DTG and MIP sample preparation and method: Samples were taken from the central part of the same specimen that had completed the 90-day shrinkage rate test. The sample mass was approximately 1 g, with a size of 3 mm × 3 mm × 3 mm. The vacuum freeze-drying method (Spanish Telstar-85 plus) was used. The drying conditions were −50 °C, vacuum degree of 10 Pa, and drying time of 24 h, until the sample mass change was less than 0.1%. Simultaneous Thermal Analysis (TG-DTG) is used to heat the sample and analyze its mineral phase composition. The experiments were carried out using the METTLER TOLEDO TGA/DSC (from Zurich, Switzerland) in a nitrogen environment with a temperature setting of 30 °C to 800 °C and a rate of 10 °C/min. The mercury intrusion pore method (MIP) experiment was performed on the US Mike 9600 instrument at 0–30,000 psi and measured the porosity and pore size of the sample. The MIP test parameter setting has the contact angle set at 140°. The surface tension of mercury at 25 °C is 480 mN/m.

In addition, the data from the MIP experiment were processed using the space-filling model [53] to determine the fractal dimension of the sample. The calculation method is expressed by the following equation:

(2) lgV=lgC+(3−D)lgφ

where *V* is the pore volume or mercury intake, *C* is a constant, *D* is the fractal dimension, and *φ* is the pore aperture.

## 3. Results and Analysis

### 3.1. Simultaneous Thermal Analysis

**Figure 5:**
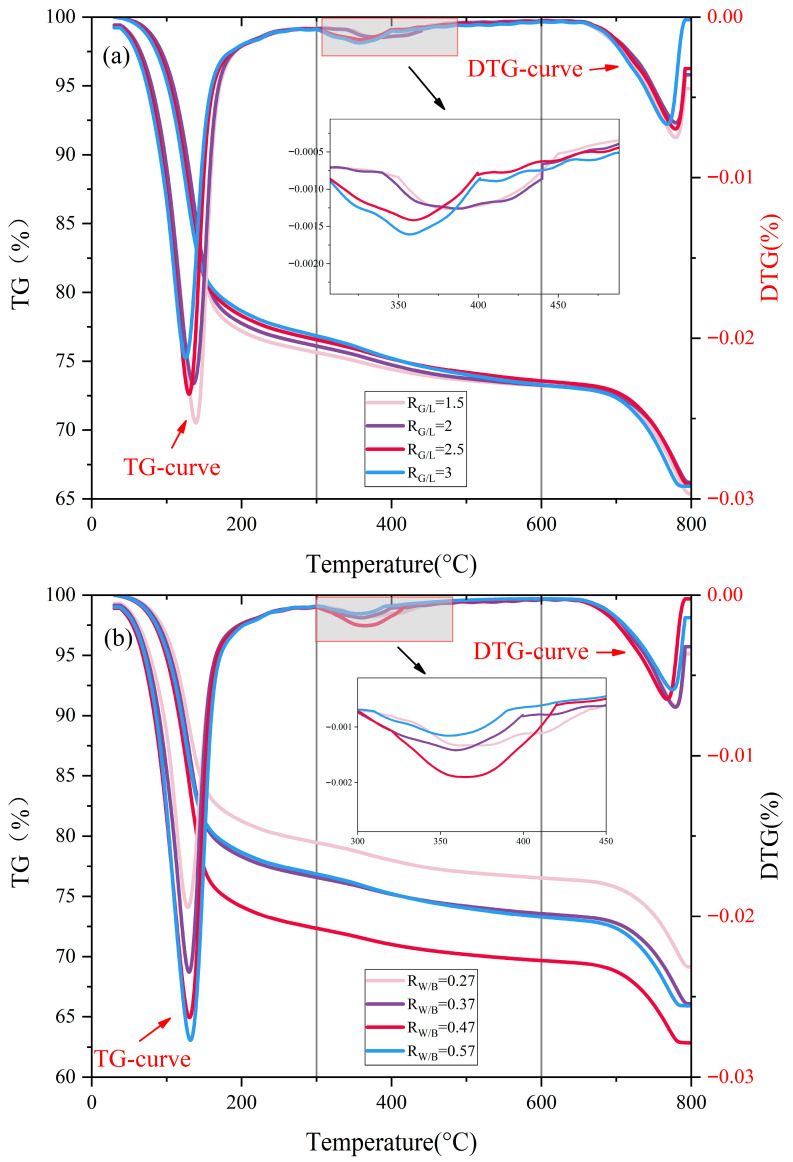
TG-DTG curves of LSGM (**a**) different *R*_G/L_ and (**b**) different *R*_W/B_.

Figure 5 shows the TG-DTG curves of LSGM with different *R*_G/L_ and *R*_W/B_ mix ratios. The TG-DTG curves of each sample have similar peak characteristics, and there are mainly three endothermic peaks and three weight loss stages. In the first stage (30–300 °C), the initial mass loss is mainly due to the volatilization of free water, which requires the absorption of energy, and the corresponding DTG curve has an obvious peak, indicating that the sample is absorbing a large amount of energy at this time. After 100 °C, the quality also decreases to a certain extent, which is due to the decomposition of a part of the bound water. In the second stage (300–600 °C), the dehydroxylation and dehydration of calcium-aluminate-silicate-hydrate (C-A-S-H gels) and sodium-aluminate-silicate-hydrate (N-A-S-H gels) occur [54,55]. In the third stage (600–800 °C), when the temperature reaches about 750 °C, the mass curve also has an obvious trend of decline, and the DTG curve also has a peak, which corresponds to the decomposition process of CaCO_3_. Given the comparable alumina content in LS and GBFS, their dissolution in an alkaline activator releases silicate and aluminate ions. These ions then compete for the available Ca^2+^ and Na^+^ to condense into C-A-S-H and N-A-S-H gels. The Ca^2+^ ions that do not participate in polycondensation can react with carbonate ions to form calcite (CaCO_3_). This interplay suggests that the content of CaCO_3_ in the LSGM can serve as an indirect indicator for estimating the proportion of C-A-S-H gels. Compared with the layered structure of C-A-S-H gels, the three-dimensional network structure of N-A-S-H gels is denser and has fewer pores.

As shown in Figure 5a, in the temperature range of 30–800 °C, the total weight loss of LSGM is positively proportional to the *R*_G/L_ ratio. Through analysis, it can be found that with the increase in the *R*_G/L_ ratio, the weight loss of the sample in the first stage begins to decrease, the weight loss of C-A-S-H gel dehydration in the second stage increases, and the content of CaCO_3_ in the third stage decreases. This indicates that with the increase in the *R*_G/L_ ratio, calcium ions in the geopolymer that originally participated in the polycondensation reaction turn into CaCO_3_. CaCO_3_ plays a positive role in improving the early strength of geopolymers. As a calcite crystal, CaCO_3_ can fill the spaces between the particles of the cementified material, and as a micro-aggregate, it helps to improve the long-term stability of the geopolymer. And the Ca^2+^ are converted into CaCO_3_, meaning that more N-A-S-H gels are generated in the polycondensation reaction.

Figure 5b shows that mass loss in the first stage increases significantly as *R*_W/B_ increases. This means that an increase in *R*_W/B_ leads to an increase in the content of free and bound water in the LSGM. In the second stage, with the increase in *R*_W/B_, the endothermic peak first increased and then decreased. This shows that an appropriate amount of water can promote the polycondensation of geopolymers. However, when the water is excessive, the concentration of the alkali activator will be diluted, and the process of polycondensation will be slowed down.

### 3.2. Pore Structure

Regarding the pore size distribution characteristics, the pores in LSGM are classified into four categories based on the established standards for alkali-activated materials and cementitious systems [56,57]. (1) Macropores (>200 nm): Mainly consisting of capillary pores formed by incomplete slurry compaction, which facilitate water migration and increase shrinkage potential. (2) Mesopores (50–200 nm): Defined as harmful pores that contribute significantly to capillary stress during water loss, exacerbating shrinkage. (3) Fine mesopores (20–50 nm): Classified as minimally harmful, as their narrow diameter reduces the rate of water evaporation and associated stress. (4) Micropores (<20 nm): Primarily gel pores within the C-A-S-H matrix, which are beneficial for reducing shrinkage due to strong water retention via chemical bonding.

**Figure 6:**
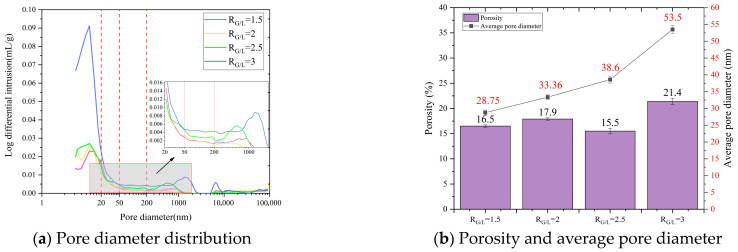

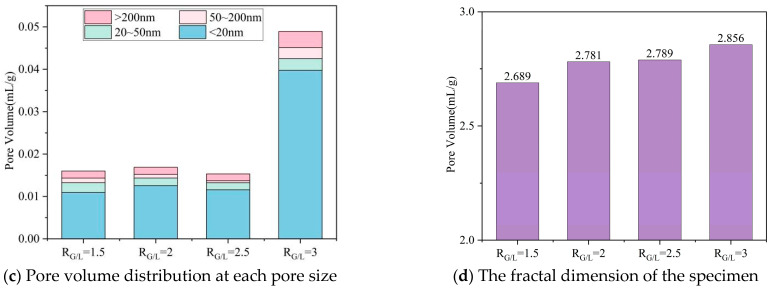
Pore characteristics of LSGM with different *R*_G/L_.

The pore structure distribution characteristics of LSGM at different *R*_G/L_ are shown in Figure 6. With the increase in *R*_G/L_, the pore size distribution curve in Figure 6a shows an upward trend, especially when *R*_G/L_ = 3. The proportion of macropores and micropores showed an increasing trend, and the proportion of mesopores increased significantly. The mean pore size and porosity of LSGM increased with the increase in *R*_G/L_. It can be seen from Figure 6b,c that the porosity, average pore size, and pore volume of the sample also increase with the increase in *R*_G/L_. The average pore size of the sample increased from 28.75 nm to 53.5 nm, and the porosity increased from 16.5% to 21.4%, with growth rates of 86% and 30%, respectively.

Based on topological theory and fractal theory, the fractal dimension of the sample can be calculated accurately. The larger the fractal dimension of the sample, the more irregular and complex the pore distribution inside the LSGM. The fractal dimension of different LSGM under the condition of *R*_G/L_ is shown in Figure 6d. It can be seen that with the increase in *R*_G/L_, the fractal dimension of LSGM also increases, and the pores in the sample become more complex and irregular. The increase in fractal dimension is roughly proportional to the volume of mesopores. Moreover, a higher fractal dimension suggests that there are more gelling materials in the LSGM, or that the gelling materials tend to be more porous.

In summary, the addition of LS makes the internal structure of the LSGM denser. Because of the low calcium characteristics of LS, the alkali activation polycondensation reaction can produce more N-A-S-H gels, thus reducing the proportion of low-density C-A-S-H gels. In addition, the unreacted LS has a filling effect on the pores of the LSGM, which can effectively fill the macropores and micropores of C-A-S-H gels and refine the overall pore size.

**Figure 7:**
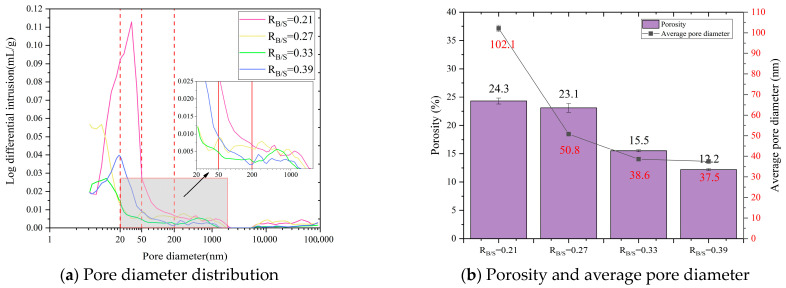

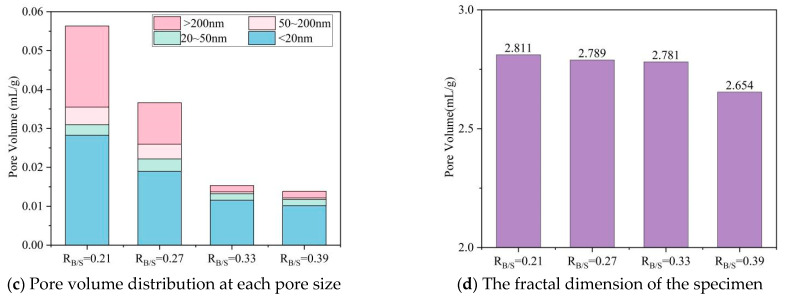
Pore characteristics of LSGM with different *R*_B/S_.

The pore structure distribution characteristics of LSGM with different RB/S are shown in Figure 7; the pore size distribution curve has a peak in the area of micropores. With the increase in *R*_B/S_, this peak begins to decrease and move to the area of micropores, and the mesoporous content also decreases. With the increase in *R*_B/S_ from 0.21 to 0.39, the porosity of the sample decreased by 50% from 24.3% to 12.2%, and the average pore diameter also decreased by 63% from 102.1 nm to 37.5 nm. In particular, the group of *R*_B/S_ = 0.21 macropores reached 0.028 mL/g, accounting for 50% of the pore volume. With the increase in RB/S, the reduction in sand content results in a rapid decrease in the number of micropores. Moreover, it can be seen from Figure 7d that the fractal dimension of the sample decreases with the increase in *R*_B/S_, and the pores inside the sample tend to become more regular.

According to the analysis, most macropores form because the gel is insufficient to coat excessive sand and gravel, leading to the formation of loose bonding surfaces and cavities, which results in unfavorable porosity, pore size, and pore size distribution of the sample. As *R*_B/S_ increases, sufficient gel can coat the sand well, thereby minimizing the formation of interfacial pores between the gel matrix and sand particles.

### 3.3. Shrinkage Strain

**Figure 8:**
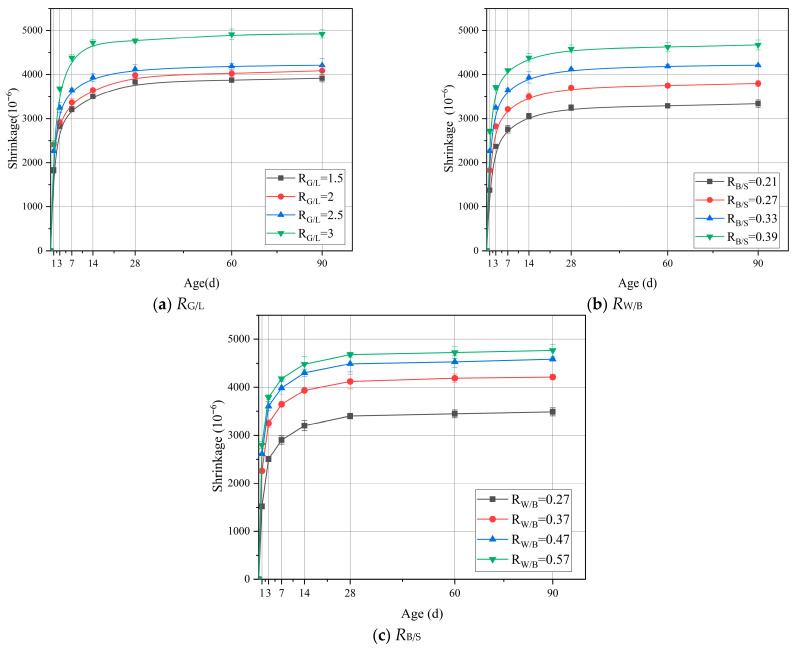
Shrinkage strain of shrinkage under different composition ratios.

Figure 8 shows the 90 d shrinkage strain performance of LSGM with different component ratios. It can be found that when *R*_G/L_ = 3, the 90 d shrinkage strain of the specimen is the highest (4928 με), which is 25.8% higher than that when *R*_G/L_ = 1.5 (3917 με). This clearly shows that reducing *R*_G/L_ can effectively reduce the porosity of the sample and thus slow down the shrinkage strain of the LSGM. The reduction in the *R*_G/L_ value facilitates the formation of N-A-S-H gel. During the curing process, due to the inherent properties of the gel, the N-A-S-H gel exhibits a more compact structure compared to C-A-S-H, with fewer pores and reduced water loss. Simultaneously, the increase in LS leads to greater calcium carbonate formation, thereby enhancing early strength. Consequently, during curing, the higher early strength and less structural alteration in pore structure effectively suppress capillary stress-induced shrinking during the curing process.

This indicates that reducing *R*_W/B_ can effectively alleviate the shrinkage of LSGM. In geopolymer systems, water molecules that do not participate in polymerization are distributed in a free state. During the curing process, driven by the humidity gradient and temperature difference, free water is transferred from the interior of the sample to the external environment. The continuous water dissipation in pores breaks the balance of internal and external pressure and causes the redistribution of pore wall stress. When water evaporates, the gas–liquid boundary recedes into the pores, and the curvature of the meniscus changes. According to Laplace’s equation, this creates negative pressure in the pores. With continuous curing, the negative pressure accumulates continuously. Once the tensile strength of the LSGM matrix is exceeded, micro-cracks will occur in the microstructure, causing deformation and damage, and the macroscopic performance shows an increase in shrinkage strain. At the same time, the concentration of the alkali activator also affects the gel density in LSGM. Within a certain concentration range, reducing the alkaline activator concentration facilitates the formation of a denser gel matrix, which effectively restricts moisture migration and evaporation, thereby mitigating shrinkage. This improvement stems from the milder alkaline environment, which enables a more controlled and gradual dissolution of the raw mineral materials. As a result, the dissolved silicate and aluminate species have sufficient time to diffuse and reorganize, ultimately leading to the development of a homogeneous gel network with refined pore structure. In contrast, increasing the activator concentration generally promotes the formation of a more porous microstructure. Under highly alkaline conditions, the excessively rapid dissolution of mineral precursors, followed by accelerated precipitation, tends to yield a disordered and loosely packed gel, resulting in a coarsened pore framework. Such a coarse microstructure offers reduced resistance to the movement of capillary water. When this free water evaporates from the interconnected capillary pores, significant tensile stresses develop, ultimately inducing pronounced shrinkage.

It can be clearly observed from Figure 8c that with the increase in *R*_B/S_, the shrinkage strain rate shows an increasing trend. When *R*_B/S_ = 0.21, the shrinkage strain is 3340 με. When *R*_B/S_ = 0.39, the shrinkage strain increased by 39.8% to 4670 με. This series of data intuitively shows that reducing the *R*_B/S_ ratio can effectively alleviate the shrinkage phenomenon. The reason is that when the *R*_B/S_ is relatively high, relatively loose bonding interfaces and voids tend to form between sand and gels. These features create a pore structure that facilitates water loss, which in turn causes capillary pressure to increase and ultimately increases shrinkage strain.

Through analysis of shrinkage strain performance and MIP experiment results, it can be clearly seen that the shrinkage value is highly consistent with the changing trend of pores, especially the proportion of mesoporous pores in the total porosity. From the data and phenomenon, the higher the proportion of pores in the total pores, the more significant the shrinkage phenomenon. This strongly indicates that the pores in the sample provide a potential path for the free volatilization of water and greatly accelerate the volatilization process of water in the sample. The rapid evaporation of water causes the pressure difference in the pores, which leads to shrinkage stress and finally intensifies the shrinkage phenomenon of LSGM.

## 4. Mathematical Prediction Model

### 4.1. Existing Mathematical Model

At present, there is a lack of accurate and effective models to predict the shrinkage of geopolymers, and most of the existing models are based on the modification of the shrinkage characteristics of cement-based materials. Common shrinkage prediction models include the ACI 209 model (American Concrete Institute Model) [58], which estimates the shrinkage rate through empirical formulas based on the basic composition and environmental factors of the concrete. The CEB90 model (European Concrete Commission 1990 model) [59] takes into account factors such as cement type, the water–cement ratio, and curing conditions and describes the shrinkage process through complex mathematical formulas. The B3 model [60] can predict the long-term shrinkage behavior of cement-based materials by thermodynamic and mechanical methods. The GL-2000 model [61], combined with the physical and chemical properties of the material and environmental conditions, has advantages in predicting shrinkage under different operating conditions. Sakata’s model [62,63] focuses on the effect of mineral composition reaction on the shrinkage of cement-based materials. As shown in Table 3, the functional form of these models is complex.

**Figure 9:**
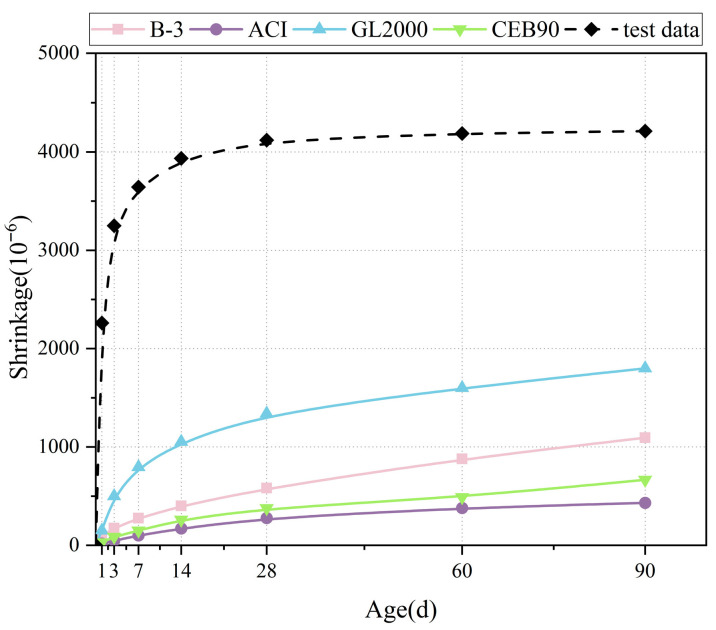
Comparison of experimental data with traditional mathematical model.

Figure 9 compares the original model curve with the test curve (*R*_G/L_ = 2.5, *R*_W/B_ = 0.47, *R*_B/S_ = 2.5). The comparison results show that there are significant differences between the calculated values of the cement-based material shrinkage prediction model and the experimental values of LSGM, and the calculated values are much smaller than the experimental values, which indicates that the model cannot accurately predict the shrinkage strain of LSGM. In addition, there are also large errors between the model and the actual test results in terms of the limiting shrinkage rate and the trend of the shrinkage rate with the growth of age, which makes it difficult to meet the actual engineering requirements. However, reconstructing a new model requires a lot of experimental data to construct semi-empirical formulas. It is feasible to use the concrete shrinkage model as the frame and the LSGM shrinkage experimental data to adjust and put forward a modified model suitable for the LSGM shrinkage prediction of lithium slag base.

### 4.2. Modified Mathematical Model

By summarizing the existing concrete shrinkage prediction models, it can be found that most of these functions are hyperbolic functions or hyperbolic power functions, and the models are composed of two parts: a basic formula and an influence coefficient. By comparing the applicable conditions and the complexity of functions, the ACI model has better expansibility, simpler function form, and more abundant variables involved in the influence coefficient. The mix ratio variable of this study can be well matched, which also leaves room for further improvement of the model at a later stage. The basic formula of the ACI model is as follows:

(3) $$\varepsilon_{sh}=\varepsilon_{sh\infty }\frac{t}{t+f}$$

(4) $$\varepsilon_{sh\infty }=780\gamma_{\mathrm{cp}}\gamma_{\lambda }\gamma_{h}\gamma_{s}\gamma_{\phi }\gamma_{a}$$

$\varepsilon_{sh}$ is the shrinkage value of the sample on day t. $\varepsilon_{sh\infty }$ is the limit shrinkage value. t indicates the age from the beginning of the test. *f* is the parameter based on the curing conditions, and γ is the related influence coefficient. $\gamma_{\mathrm{cp}}$ is the correction coefficient for curing conditions, $\gamma_{\lambda }$ is the correction coefficient for environmental relative humidity, $\gamma_{h}$ is the correction coefficient for sample thickness, $\gamma_{s}$ is the correction coefficient for slump, $\gamma_{\phi }$ is the correction coefficient for the sand ratio, and $\gamma_{a}$ is the correction coefficient for cementitious material content.

In traditional models, the final shrinkage value of cement-based materials is generally determined by concrete strength and the related influence coefficient, but there are many factors affecting the strength of geopolymers. In addition, different blending ratios have different effects on the strength and shrinkage rate of mortar. Therefore, *R*_G/L_, *R*_W/B_, and *R*_B/S_ are used to predict the final shrinkage rate of LSGM, which makes the model more concise and directly reflects the relationship between variables and results. Based on the experimental data in Section 3, nonlinear curve fitting is carried out by Origin software with Formula (3) as the framework. Formulas (5) and (6) are as follows:

(5) $$\varepsilon_{sh\infty }=3915\cdot \alpha_{R_{G/L}}\cdot \alpha_{R_{W/B}}\cdot \alpha_{R_{B/S}}$$

(6) $$\beta =1.203\cdot \beta_{R_{G/L}}\cdot \beta_{R_{W/B}}\cdot \beta_{R_{B/S}}$$

where $\alpha_{R_{G/L}},\alpha_{R_{W/B}},\alpha_{R_{B/S}}$ are the influence coefficients of variables *R*_G/L_, *R*_W/B_, and *R*_B/S_ on parameter $\varepsilon_{sh\infty }$, respectively. $\beta_{R_{G/L}},\beta_{R_{W/B}},\beta_{R_{B/S}}$ are the influence coefficients of variables *R*_G/L_, *R*_W/B_, and *R*_B/S_ on parameter β, respectively.

Since different metrics have varying units and numerical ranges, direct analysis may result in weight loss due to dimensionality issues. To eliminate interference from data dimension differences during analysis, we first normalize the collected data by mapping all values to the 0–1 range. Subsequently, we perform individual fitting of these influence coefficients based on this standardized framework. According to the results, we successfully derived the specific expressions of parameters $\varepsilon_{sh\infty }$ and β, as shown below.

(7) $$\varepsilon_{sh}=\varepsilon_{sh\infty }\frac{t}{t+\beta }$$

(8) $$\varepsilon_{sh\infty }=3915\cdot \alpha_{R_{G/L}}\cdot \alpha_{R_{W/B}}\cdot \alpha_{R_{B/S}}$$

(9) $$\beta =1.203\cdot \beta_{R_{G/L}}\cdot \beta_{R_{W/B}}\cdot \beta_{R_{B/S}}$$

(10) $$\alpha_{R_{G/L}}=1.488-0.568R_{G/L}+{0.165R_{G/L}}^{2}$$

(11) $$\alpha_{R_{W/B}}=0.107+3.45R_{W/B}-{2.99R_{W/B}}^{\;2}$$

(12) $$\alpha_{R_{B/S}}=0.453+1.68R_{B/S}$$

(13) $$\beta_{R_{G/L}}=1.48-0.29R_{G/L}$$

(14) $$\beta_{R_{W/B}}=3.44-10.09R_{W/B}+{9.66R_{W/B}}^{\;2}$$

(15) $$\beta_{R_{B/S}}=2.25-3.84R_{B/S}$$

where t is the number of maintenance days, $\varepsilon_{sh}$ is the shrinkage strain of any number of days, $\varepsilon_{sh\infty }$ is the ultimate shrinkage strain, *R*_G/L_ is the GBFS-LS ratio, *R*_W/B_ is the water–binder ratio, and *R*_B/S_ is the binder–sand ratio.

### 4.3. Model Verification

**Figure 10:**
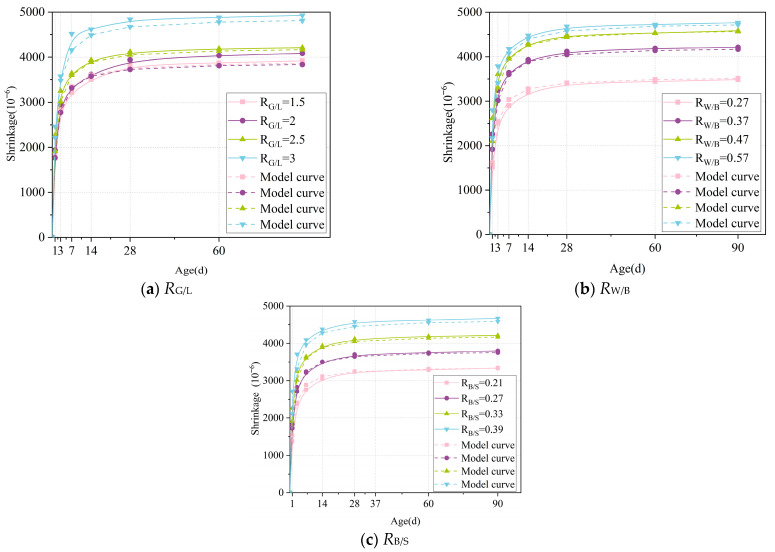
Comparison of experimental data with Shrinkage Prediction model.

Firstly, the limit shrinkage rate ($\varepsilon_{sh\infty }$) of the sample was accurately calculated according to the model. Then, with the help of the time parameter, the shrinkage value of LSGM on different days was calculated. The relevant results are shown in Figure 10, from which it can be intuitively seen that the model curve proposed in this paper fits well with the actual test data curve. Through further analysis of the determination coefficient *R*^2^, the obtained results are all greater than 0.98, which strongly proves that the fitting between the model and the test data is ideal. The high degree of fitting means that the model can accurately describe the shrinkage behavior of the LSGM during the process, which provides a valuable reference for the optimization and expansion of the performance of the LSGM.

## 5. Conclusions

In the study of LSGM, the effects of different mix ratios on its mineral composition, pore structure, and shrinkage characteristics were analyzed. Through experimental exploration and data analysis, the following main conclusions are drawn:

(1) Compared with the sample (RG/L), the low calcium property of LS alters the polymerization path of silicate ions: it reduces the concentration of Ca2+ in the system, promoting the polymerization of Na+ with silicate to form a high-density N-A-S-H gel. At the same time, the unreacted LS particles can fill the 50–100 nm micropores of the C-A-S-H gel, reducing the average pore diameter from 53.5 nm to 28.75 nm, further blocking the capillary water migration channels.
(2) When the water–binder ratio decreases from 0.57 to 0.27, the 90-day shrinkage reduces by 36.7%. The MIP test shows that at this time, the proportion of mesopores increases by 27.6%, the capillary stress caused by the evaporation of free water significantly increases, and the shrinkage strain is 36.7% higher than that when RW/B = 0.37; when RW/B < 0.27, insufficient fluidity of the slurry occurs, resulting in decreased vibration compactness, and causes a slight increase in the proportion of harmful pores.
(3) The ratio of RB/S has a significant impact on the performance of LSGM, with the core mechanism being that the integrity of cementitious material coating sand particles determines interfacial porosity. The binder–sand ratio increased from 0.39 to 0.21; the 90-day shrinkage rate decreased by 39.8%. This is because at a low RB/S ratio, there is sufficient cementitious material to fully encapsulate the sand particles, optimize the transition zone between sand and cement, and reduce loose contact surfaces and voids.
(4) Based on a thorough consideration of the characteristics of LSGM and combined with experimental data, the influence coefficients of the GBFS-LS ratio (RG/L), water–binder ratio (RW/B), and cement–sand ratio (RB/S) were introduced into the ACI model framework to construct the LSGM shrinkage prediction model. The fitting goodness R2 of this model is greater than 0.98, and its adaptability to different mix ratios is significantly better than the traditional model. This model provides a quantitative tool for LSGM engineering design, which can significantly reduce the trial-making cost and cycle.

## Figures and Tables

**Table 1:** Raw material component properties.

Raw Material		GBFS	LS
Chemical composites (%)	SiO_2_	34.2	39.8
	CaO	34	16.9
	Al_2_O_3_	17.6	15.3
	Fe_2_O_3_	1.01	3.98
	MgO	6.21	1.40
	SO_3_	1.62	9.74
	Li_2_O	/	0.26
	Other	5.36	12.62

**Table 2:** The mix ratio of the LSGM sample.

No	Samples	GBFS (g)	LS (g)	Sand (g)	Water (g)
1	*R*_G/L_-1.5	900	600	495	555
2	*R*_G/L_-2	1000	500	495	555
3	*R*_G/L_-2.5	1072	428	495	555
4	*R*_G/L_-3	1125	375	495	555
5	*R*_B/S_-0.21	1072	428	315	555
6	*R*_B/S_-0.27	1072	428	405	555
7	*R*_B/S_-0.33	1072	428	495	555
8	*R*_B/S_-0.39	1072	428	585	555
9	*R*_W/B_-0.27	1072	428	495	405
10	*R*_W/B_-0.37	1072	428	495	555
11	*R*_W/B_-0.47	1072	428	495	705
12	*R*_W/B_-0.57	1072	428	495	855

**Table 3:** The functional form of a shrinkage prediction model for cement-based materials.

Model	Functional Form	
ACI	$\varepsilon_{\mathrm{sh}}(t,t_{sho})=\frac{{\left(t-t_{sho}\right)}^{\alpha }}{f+(t-t_{sho})}{\varepsilon_{sh}}_{\infty }$	$\varepsilon_{\mathrm{sh}}(t,t_{sho})=\frac{{\left(t-t_{sho}\right)}^{\alpha }}{35+(t-t_{sho})}{\varepsilon_{sh}}_{\infty }(\mathrm{moist}/\mathrm{cure})$
CEB-90	$\varepsilon_{\mathrm{sh}}=\varepsilon_{shu}\beta (h)\beta (t)$	$\varepsilon_{shu}=1000K\;{\left(\frac{4350}{{f^{\prime }}_{cm28}}\right)}^{1/2}10^{-6}$
B3	$\varepsilon_{\mathrm{sh}}(t,t_{0})=-{\varepsilon_{sh}}_{\infty }K_{h}S(t)$	${\varepsilon_{\mathrm{sh}}}_{\infty }=-\alpha_{1}\alpha_{2}(26(w{)}^{2.1}{\left(f{’}_{c}\right)}^{-0.28}+270)10^{-6}$
Gardner	$\varepsilon_{\mathrm{sh}}=\varepsilon_{shu}\beta (h)\beta (t)$	$\varepsilon_{shu}=1000K\;{\left(\frac{4350}{{f^{\prime }}_{cm28}}\right)}^{1/2}10^{-6}$
Sakata	$\varepsilon_{\mathrm{sh}}(t,t_{0})=\varepsilon_{sh\infty }(1-\exp\{-0.108{\left(t-t_{0}\right)}^{0.56})$	$\begin{array}{c} \varepsilon_{sh\infty }=-50+78(1-\exp(\mathrm{RH}/100)+38(\ln(w))\\ -5{\left(\ln(V/S/10)\right)}^{2}10^{-5} \end{array}$

where *ε*_sh_ is the shrinkage strain; *ε*_sh∞_ and *ε_shu_* are the ultimate shrinkage strain; *β*(h) is the correction term for the humidity impact factor; β(t) is the correction term of time impact factor; t is the age of the geopolymer mortar; t_0_ is the age of the geopolymer mortar at the start of drying; *f*’_cm28_ represents the compressive strength after 28 days of curing; *t*_s_ is the curing age of the geopolymer mortar; *ε*_sh,0_ is the nominal shrinkage strain; and *h* is the effective cross-section thickness.

## Data Availability

The original contributions presented in this study are included in the article. Further inquiries can be directed to the corresponding authors.
